# Genetics of Obesity in East Asians

**DOI:** 10.3389/fgene.2020.575049

**Published:** 2020-10-20

**Authors:** Chang Sun, Peter Kovacs, Esther Guiu-Jurado

**Affiliations:** Medical Department III – Endocrinology, Nephrology, Rheumatology, University of Leipzig Medical Center, Leipzig, Germany

**Keywords:** genetics, GWAS, obesity, BMI, East Asians

## Abstract

Obesity has become a public health problem worldwide. Compared with Europe, people in Asia tend to suffer from type 2 diabetes with a lower body mass index (BMI). Genome-wide association studies (GWASs) have identified over 750 loci associated with obesity. Although the majority of GWAS results were conducted in individuals of European ancestry, a recent GWAS in individuals of Asian ancestry has made a significant contribution to the identification of obesity susceptibility loci. Indeed, owing to the multifactorial character of obesity with a strong environmental component, the revealed loci may have distinct contributions in different ancestral genetic backgrounds and in different environments as presented through diet and exercise among other factors. Uncovering novel, yet unrevealed genes in non-European ancestries may further contribute to explaining the missing heritability for BMI. In this review, we aimed to summarize recent advances in obesity genetics in individuals of Asian ancestry. We therefore compared proposed mechanisms underlying susceptibility loci for obesity associated with individuals of European and Asian ancestries and discussed whether known genetic variants might explain ethnic differences in obesity risk. We further acknowledged that GWAS implemented in individuals of Asian ancestries have not only validated the potential role of previously specified obesity susceptibility loci but also exposed novel ones, which have been missed in the initial genetic studies in individuals of European ancestries. Thus, multi-ethnic studies have a great potential not only to contribute to a better understanding of the complex etiology of human obesity but also potentially of ethnic differences in the prevalence of obesity, which may ultimately pave new avenues in more targeted and personalized obesity treatments.

## Introduction

Obesity has become a public health problem throughout the world, whether in developing or developed countries (Ng et al., 2014), and is well recognized as a risk factor for a wide variety of health problems such as diabetes, dyslipidemia, hypertension, and cardiovascular diseases (Van Gaal et al., 2006). Along with the generally acknowledged role of environmental factors such as sedentary lifestyle combined with the intake of energy-dense nutrition and insufficient energy expenditure, development of obesity likely also has a genetic component as demonstrated by both monogenic and common polygenic forms of obesity. Early data from white male twins (Stunkard, 1986), Quebec inhabitants (Bouchard et al., 1990), French family (Pérusse et al., 1996), and Danish adoption studies (Stunkard et al., 1986) showed that obesity and fat distribution have a strong genetic susceptibility, with heritability estimates ranging from 40 to 70% for obesity risk and from 36 to 61% for waist-to-hip ratio (WHR) (Stunkard, 1986; Bouchard et al., 1990). It is important to note that the measured heritability depends on the environmental variance in the study population, which if low, can overestimate heritability. Recent research efforts in ethnically diverse individuals highlighted the genetic contribution even for changes in body weight after interventions such as metabolic surgeries (Fesinmeyer et al., 2013; Parikh et al., 2013). Although body mass index (BMI) is a standard measure of obesity, WHR reflecting central body fat distribution is the main predictor of obesity-related metabolic sequelae such as type 2 diabetes (T2D) or cardiovascular diseases (Manolopoulos et al., 2010). Being aware of the significant health burden associated with obesity, a better understanding of its complex pathophysiology including the genetic component remains a major challenge of the current obesity research.

Recent advances in high-throughput genotyping technologies allowed the development of powerful analytical tools like genome-wide association studies (GWASs) to explore novel genes and loci contributing to the genetic susceptibility of complex diseases. In the past decade, large-scale GWASs uncovered hundreds of genetic risk loci for BMI and WHR in European populations, making remarkable progress in our understanding of the genetics of these complex polygenic traits. A large meta-analysis of GWAS for BMI in ∼700,000 European individuals revealed over 750 BMI-associated single-nucleotide polymorphisms (SNPs), although only explaining 6.0% of the BMI variance (Yengo et al., 2018). The vast majority of BMI and obesity loci map to the non-coding genome; therefore, GWASs for the vast majority of cases do not usually identify specific genes but only susceptibility regions in the genome. Converting genetic risk loci into effector transcripts and function has been slow and has become a field of research in itself. Also, imperfect correlation between effect sizes measured from a population-based approach such as GWAS and effect sizes measured from a sibling approach suggests potentially a significant amount of indirect genetic effects (genetic nurture) captured in GWAS “direct” effects for a trait like BMI (Young et al., 2020). It is also noteworthy that currently established methods such as Genome-wide Polygenic Score (GPS), which estimates heritability of human complex traits in unrelated individuals using whole-genome sequencing data, further evidenced a missing heritability in BMI compared with assumptions based on earlier studies (Khera et al., 2019). Based on the fact that most of the previously reported GWAS were done in individuals of European ancestry, non-European ancestries may provide an attractive and very promising source for upcoming genetic studies aimed at identification of novel proposed mechanisms underlying the associations of genetic loci with obesity. Although these studies may contribute to a better understanding of the genetics of obesity and to decreasing the proportion of the missing heritability, there are several specific features which have to be considered in multi-ethnic genetic analyses. Due to the fact that the multifactorial character of obesity includes both genetics and a strong environmental component, the specified loci may have distinct contributions in different ancestral genetic backgrounds and different environments as presented through diet and exercise among other factors. An epidemiological survey found that people in Asia have lower obesity rates compared with people in Europe and the United States, but type 2 diabetes is more prevalent in Asia even with lower BMI (Yoon et al., 2006).

In the case of the United States, obesity rates also vary by ethnic groups or social classifications of race. Only 4.8% of Asian Americans (including Chinese, Japanese, Korean, Asian Indian, Vietnamese, Filipino, and others) over the age of 30 had obesity between 2001 and 2002. The prevalence was lower than in other ethnic groups (21.8% of European Americans, 34.8% of African Americans) living in the United States (Wang and Beydoun, 2007). The National Center for Health Statistics reported that the prevalence of obesity was lowest among non-Hispanic Asian adults (11.7%) and youth (8.6%), followed by non-Hispanic white (34.5%, 14.7%), Hispanic (42.5%, 21.9%), and non-Hispanic black (48.1%, 19.5%) in the United States between 2011 and 2014 (Ogden et al., 2015). A recent study by Commodore-Mensah et al. (2018), also confirmed the lowest prevalence of overweight/obesity in Asians, even after adjusting for WHO-recommended Asian-specific BMI cutoffs (overweight: 23–27.4 kg/m^2^; obesity: ≥27.5 kg/m^2^) in the United States between 2010 and 2016. The reason for these disparities are multifactorial including lifestyle, health care, income status, experience of discrimination, and changes in diet after migration to the United States; however, these data may also implicate the role of genetics and interactions between genetics and the environment in creating variation in obesity rates. Even though highly challenging, investigations of the genetic and environmental factors underlying variation in the pathophysiology of obesity are highly desirable as they could ultimately lead to improved knowledge of the causal mechanistic chains underlying the pathophysiology of this disease and its related metabolic sequelae.

Currently, there is increasing evidence indicating the potential role of genetic ancestry in variable predisposition to obesity in different environments. This review provides a comprehensive overview of recent advances in obesity genetics in individuals of Asian ancestry. In particular, we compared obesity susceptibility loci discovered in individuals of European and Asian ancestries and addressed the potential role of genetic variants in variation in obesity risk.

It has to be acknowledged that the information on ancestry variables in GWASs is commonly based on self-reported questionnaires. This practical way to adjust for ancestry in genetic association studies has been previously certificated to be sufficiently accurate for assessing population stratification in genetic association studies (Rosenberg et al., 2002). However, it may be misleading in comparisons of complex traits across populations and may overestimate polygenic adaptation due to residual population stratification (Sohail et al., 2019). Despite strong associations reaching *p*-values with genome-wide statistical significance, these analyses may be all subtly affected by population structure, leading to partly incorrect effect estimations (Sohail et al., 2019). Differences in genetic structure among populations are mostly due to genetic drift, natural selection, *de novo* mutations, and admixture.

Although this review focuses on East Asian populations, no ancestry (geographic) region can be considered in isolation in terms of human population history because migrations between Asians and Europeans have had a substantial impact on current genetic structure. For instance, the ancient DNA studies showed that most present-day Europeans derive from at least three highly differentiated populations: west European hunter-gatherers, ancient north Eurasians from the Steppe, and early farmers from Anatolia, and that there are varying proportions of these different ancestries across Europe. In early Bronze Age pastoralists, West Eurasian ancestry and East Asian ancestry have already undergone genomic mixture through the Eurasian steppes (Mathieson et al., 2015; Damgaard et al., 2018). There is still a controversy about the genetic gradients in present-day Asians, where the ancestry variables may be more complicated and diverse. There are at least three genetic gradients in the South Asian region: Anatolian/Iranian farmer-related ancestry, Ancestral North Indians, and Ancestral South Indians who were mixed with northwestern and southeastern groups with Steppe ancestry (Narasimhan et al., 2019); at least four ancient populations in Southeast Asia: mainland Hoabinhians, Andamanese onge, Malaysian jehai, and ancient Japanese Ikawazu Jomon (Mccoll et al., 2018); and at least three ancient populations in East Asia (e.g., Japanese): Hondo, Ryukyu, and Ainu (Takeuchi et al., 2017).

We have to admit that although these aspects are not addressed in our review, the readers should be aware of them. Also, we do not address the diversity among South Asians, Southeast Asians, and East Asians, which is based on the following two points: (1) in 13 BMI-related genetic Asian studies (Table 1), only two studies included South Asians and South East Asians. In addition, the sample size was strongly limited as compared with East Asians (totally: East Asian: 483,795; South Asian and South East Asian: 12,033), which did not allow a valid comparison and drawing competent and robust conclusions; (2) although South Asians may appear closer to Europeans than East Asians from the genetic point of view [e.g., there are no observed systematic differences in risk allele frequencies of WHR-related loci between South Asians and Europeans (Scott et al., 2016)], the BMI and the degree of abdominal obesity and the risk of diabetes in South Asians are comparable with East Asians (Nanditha et al., 2016).

**TABLE 1:** Studies conducted in cohorts of Asian ancestry.

**Study type**	**Publication year**	**Sample size**	**Male/female**	**Cohort age Mean (SD)**	**Criteria for discovery^c^**	**Number of variants^d^ (discovery stage)**	**Criteria for replication^e^**	**Number of variants^f^ (replication stage)**	**Number of variants replicated from^h^**	**Number of variants successfully replicated from^h^**	**References**
Candidate gene association study	1999	2056 East Asian	2056/0	24 (6)	NA	NA	*p* < 5.0E−2	NA	1	1	Siffert et al. (1999)
Candidate gene association study	2001	208 East Asian	118/90	50.2 (1.2)	NA	NA	*p* < 5.0E−2	NA	1	1	Ohshiro et al. (2001)
Candidate gene association study	2006	251 East Asian	251/0	25.5 (3.5)	NA	NA	*p* < 5.0E−2	NA	1	1	Nakano et al. (2006)
Candidate gene association study	2006	408 East Asian	135/273	59.4 (13.2)	NA	NA	*p* < 5.0E−2	NA	2	1	Wang et al. (2006)
GWAS	2009	16,703 (Dis^a^: 8842 East Asian; Rep^b^: 7861 East Asian)	7397/9306	54.4 (8.4)	*p* < 1.0E−5	2	*p* < 5.0E−2	1	NA	NA	Cho et al. (2009)
Fine mapping *FTO* study	2008	2427 East Asian	1077/1350	48.7 (15.4)	MAF > 10%	90	*p* < 1.7E−4	15	NA	NA	Hotta et al. (2008)
Replication study	2009	2865 East Asian	1420/1445	49 (14.7)	NA	NA	*p* < 5.0E−2	NA	27	11	Hotta et al. (2009)
Replication study	2010	7705 East Asian	3511/4194	49 (11.9)	NA	NA	*p* < 5.0E−2	NA	14	4	Ng et al. (2010)
Meta-analysis-GWAS	2012	83,048 (Dis: 22,762 East Asian, 4953 South and East Asian; Rep: 2118 South East Asian, 53,215 East Asian)	34,906/48,142	55.2 (9.9)	*p* < 1.0E−4	848	*p* < 5.0E−7	7	NA	NA	Wen et al. (2012)
GWAS	2012	10,391 (2431 South East Asian, 5429 East Asian, 2531 South Asian); 1006 (1006 Chinese)	5185/5297; 512/492	57.3 (10.6); 9	*p* < 5.0E−2	31	*p* < 5.0E−2	13	NA	NA	Dorajoo et al. (2012)
GWAS	2012	62,245 (Dis^a^: 26,620 East Asian Rep^b^: 35,625 East Asian)	35,870/26,375	58 (12.1)	*p* < 5.0E−5	36	*p* < 5.0E−8	7	NA	NA	Okada et al. (2012)
Meta-analysis-GWAS	2014	134,091 (Dis^a^: 86,739 East Asian, 4301 South East Asian; Rep^b^: 47,352 East Asian)	60,628/73,463	55.4 (9.8)	*p* < 7.59E−6	8	*p* < 5.0E−2	4	55	26^g^	Wen et al. (2014)
GWAS	2017	173,430 (Dis^a^: 158,284 East Asian; Rep^b^: 15,146 East Asian, 322,154 European)	90,992/82,438	59.1 (11)	*p* < 5.0E−8; *p* < 1.0E−6	72; 134	*p* < 5.0E−8	85 (51 novel)	163	66	Akiyama et al. (2017)

*^a^Discovery stage sample. ^b^Replication stage sample. ^c^Criteria for discovery stage. ^d^Number of variants found in the discovery stage by ^c^ criteria. ^e^Criteria for replication in Asian study (also as a criteria for other type studies). ^f^ Number of variants found in the replication stage by ^e^ criteria. ^g^26 variants replicated by *p* < 1E−3 from 55 variants which were identified in previous European GWASs. ^h^European GWAS (Frayling et al., 2007; Loos et al., 2008; Thorleifsson et al., 2009; Willer et al., 2009; Speliotes et al., 2010; Wen et al., 2012; Berndt et al., 2003; Guo et al., 2013; Locke et al., 2015; Shungin et al., 2015; Winkler et al., 2015). NA, not applicable.*

## Genetic Studies of Obesity Before the GWAS Era

In the last two decades of the past century, physiologic (candidate) gene association studies and genome-wide linkage studies represented the major analytical tools employed in the identification of genetic determinants of complex polygenic traits. The success of these strategies was mostly limited by poor statistical power due to the small sample sizes of the studied cohorts. Whereas linkage studies turned out to be powerful in the identification of genes responsible for monogenic Mendelian traits and diseases, their impact on polygenic traits was rather moderate. In 1999, the 825 T polymorphism in the G Protein Subunit Beta 3 gene (*GNB3*) was found as one of the first BMI-related variants in Asian ancestry individuals and replicated afterward in cohorts of European and African ancestry (Siffert et al., 1999) (Table 1). Although *GNB3* polymorphisms were not associated with BMI in a Japanese cohort (Ohshiro et al., 2001), a recent large-scale multi-population meta-analysis disclosed associations of genetic variants in *GNB3* with being overweight/obese (Li et al., 2016). In 2005, a meta-analysis containing five genome-wide linkage scan studies provided significant evidence for the association of genetic variation in the lipoprotein lipase (*LPL*) and adrenoceptor beta 3 (*ADRB3*) with BMI (Johnson et al., 2005). Subsequently, in 2007, a larger well-powered genome scan meta-analysis summarized previous genome-wide linkage scans in individuals of European ancestry (Saunders et al., 2007). Although it has not explicitly shown specific loci associated with BMI or obesity, one of the strongest candidates was the FTO alpha-ketoglutarate dependent dioxygenase (*FTO*) locus along with uncoupling protein 1 (*UCP1*), leptin (*LEP*), insulin-like growth factor 1 (*IGF-I*), scavenger receptor class B member 1 (*SCARB1*), and insulin receptor substrate 2 (*IRS2*). It should be mentioned that associations of genetic variants in *UCP1* and *LEP* with obesity have further been replicated in cohorts of Asian ancestry (Nakano et al., 2006; Wang et al., 2006).

## GWAS for BMI in Asian Populations

Within the last decade, GWAS has emerged as a powerful tool to identify loci associated with complex polygenic diseases such as obesity. As yet, GWAS contributed to the identification of more than 750 loci reaching associations with BMI at the genome-wide significance level (*p* < 10^–8^) (Yengo et al., 2018). Whereas most of the GWASs have been performed in cohorts of European ancestry (Loos et al., 2008; Thorleifsson et al., 2009; Speliotes et al., 2010; Pei et al., 2014; Locke et al., 2015; Winkler et al., 2015; Wood et al., 2016; Graff et al., 2017; Hoffmann et al., 2018; Riveros-Mckay et al., 2019), similar studies in cohorts of Asian ancestry were rather scarce. Table 2 summarizes current obesity susceptibility loci exclusively associated with cohorts of Asian ancestry.

**TABLE 2:** Obesity susceptibility loci identified (*p* < 5.0E*−*8) in cohorts of Asian ancestry.

**SNP**	**Candidate gene(s)^a^**	**Chr.^b^**	**Allele^c^ ALT/REF^d^**	**RAF^e, f^**	**Beta-estimates^c^ (SE)^e^**	***p*-value**	**References**	**Explained variance (%)^f^**
								
rs2237892	*KCNQ1*	11	T/C	0.355	0.0298 (0.0042)	9.29E−13	Wen et al. (2014)	0.041
rs671	*ALDH2*	12	A/G	0.267	−0.0378 (0.0057)	3.40E−13	Wen et al. (2014)	0.056
rs12229654	*MYL2*	12	G/T	0.224	−0.0341 (0.0058)	4.56E−09	Wen et al. (2014)	0.040
rs2076463	*FGR,IFI6*	1	G/A	0.343	−0.023 (0.004)	1.68E−08	Akiyama et al. (2017)	0.024
rs77489951	*LOC101929596,HNRNPLL*	2	T/C	0.06	0.044 (0.008)	9.39E−09	Akiyama et al. (2017)	0.022
rs8192473	*CCK*	3	T/C	0.109	−0.035 (0.006)	3.58E−09	Akiyama et al. (2017)	0.024
rs4308481	*PRDM6,CEP120*	5	C/T	0.398	0.021 (0.008)	1.00E−18	Akiyama et al. (2017)	0.021
rs183975233	*HLA-DRA,HLA-DRB5*	6	T/A	0.689	−0.031 (0.004)	7.51E−16	Akiyama et al. (2017)	0.041
rs148546399	*EYS*	6	A/G	0.01	0.050 (0.008)	1.13E−09	Akiyama et al. (2017)	0.005
rs143665886	*LINC01392,TFEC*	7	C/T	0.4	0.022 (0.004)	9.46E−09	Akiyama et al. (2017)	0.023
rs10868215	*SLC28A3,NTRK2*	9	C/T	0.299	−0.021 (0.004)	1.34E−08	Akiyama et al. (2017)	0.018
rs10795945	*CDC123,CAMK1D*	10	C/T	0.461	0.021 (0.003)	1.10E−09	Akiyama et al. (2017)	0.022
rs80117551	*HERC4*	10	C/T	0.679	−0.022 (0.004)	1.57E−08	Akiyama et al. (2017)	0.021
rs12569457	*FRAT2,RRP12*	10	T/C	0.122	0.025 (0.004)	6.67E−09	Akiyama et al. (2017)	0.013
rs1907240	*MIR5694,FGFR2*	10	G/A	0.393	−0.024 (0.004)	3.47E−11	Akiyama et al. (2017)	0.027
rs80234489	*FAM60A*	12	A/C	0.812	−0.031 (0.005)	1.05E−11	Akiyama et al. (2017)	0.029
rs75766425	*NID2*	14	C/G	0.105	0.034 (0.005)	1.28E−10	Akiyama et al. (2017)	0.022
rs4788694	*ZFHX3*	16	C/G	0.179	−0.021 (0.004)	2.54E−08	Akiyama et al. (2017)	0.013
rs180950758	*SUZ12P1*	17	T/A	0.11	0.027 (0.005)	2.63E−08	Akiyama et al. (2017)	0.014
rs1379871	*DMD*	X	C/G	0.68	0.018 (0.003)	1.05E−08	Akiyama et al. (2017)	0.014
rs6529684	*HSD17B10,HUWE1*	X	G/A	0.54	0.016 (0.003)	2.78E−08	Akiyama et al. (2017)	0.013
rs3121672	*IL13RA1*	X	C/T	0.43	0.024 (0.003)	2.90E−17	Akiyama et al. (2017)	0.028
rs1190736	*GPR101*	X	C/A	0.65	−0.017 (0.003)	1.31E−08	Akiyama et al. (2017)	0.013
rs5945324	*FAM58A,DUSP9*	X	C/G	0.4	0.022 (0.003)	1.33E−11	Akiyama et al. (2017)	0.023
rs2206271	*TFAP2B*	6	A/T	0.365	0.031 (0.008)	3.00E−18	Akiyama et al. (2017)	0.045
rs2495707	*HIF1AN,PAX2*	10	A/G	0.549	0.025 (0.008)	1.00E−09	Akiyama et al. (2017)	0.031
rs60808706	*KCNQ1*	11	A/G	0.369	0.046 (0.004)	1.24E−38	Akiyama et al. (2017)	0.099
rs3205718	*FAIM2*	12	T/C	0.231	0.023 (0.008)	4.62E−10	Akiyama et al. (2017)	0.019
rs7305242	*ALDH2,MAPKAPK5-AS1*	12	C/T	0.576	−0.021 (0.004)	2.21E−08	Akiyama et al. (2017)	0.022
rs2540034	*ADCY9*	16	T/C	0.312	0.028 (0.004)	2.97E−12	Akiyama et al. (2017)	0.034
rs35560038	*GIPR,QPCTL*	21	A/T	0.532	0.054 (0.008)	3.00E−52	Akiyama et al. (2017)	0.145

*^a^Predicted gene reported in reference studies. ^b^Chromosomes based on NCBI Build154 (GRCh38). ^c^Alternative alleles were treated as effective allele. ^d^The allele frequency based on genome Aggregation Database (gnomAD). ^e^“Standard error” according to reference studies reported. ^f^“Explained variance” is the variance explained by each reported variant using the formula which uses the allele frequency (*f*) estimated in GWAS and estimates of the additive effect (β) in meta-analysis: explained variance β^2^(1−f)2f (Akiyama et al., 2017). To estimate the additive explained variance of 31 newly identified BMI loci in Asian population, the explained variance of each individual variant were summed up and resulted in a total of 0.926%.*

In 2009, Cho et al. (2009) reported the first large-scale two-stage GWAS for quantitative traits such as BMI and height in cohorts of East Asian ancestry. The study showed that the *FTO* gene locus, which has been well acknowledged as the major contributor to polygenic obesity in European populations (Frayling et al., 2007), also provided the most prominent association signal in East Asian cohorts. Further support came from Hotta et al. (2008) who found *FTO* variant rs1558902 significantly associated with obesity in a Japanese cohort as well. It should be pointed out that *FTO* variants have not been related to obesity and being overweight only in European and Asian but also in African (Monda et al., 2013), Hispanic (Villalobos-Comparán et al., 2008; Dong et al., 2011), and Native American populations (Rong et al., 2009), in both adults and children (Dina et al., 2007; Frayling et al., 2007), implicating the global impact of *FTO* polymorphisms on obesity. *FTO* is encoding FTO alpha-ketoglutarate-dependent dioxygenase and is widely expressed in multiple tissues throughout the body, in particular, in the thalamic arcuate nucleus with the central role in body weight regulation (Gerken et al., 2007). It should be recognized that the mechanistic basis for the *FTO*-related association with obesity has been finally explained in 2015 by Claussnitzer et al. (2015). The authors showed that the functional *FTO* variant disrupted an evolutionarily conserved motif of AT-Rich Interaction Domain 5B (ARID5B) repressor, which leads to the loss of binding, releases of a potent preadipocyte super-enhancer, and activation of downstream targets Iroquois Homeobox 3 and 5 (IRX3 and IRX5) (Claussnitzer et al., 2015). This results in alterations of mechanisms controlling the shift from white adipocyte browning to lipid-storage gene expression programs, repression of basal mitochondrial respiration, decrease in thermogenesis in response to stimulus, and increase in adipocyte size, which ultimately results in human obesity (Claussnitzer et al., 2015).

Hotta et al. (2009) reported the first Japanese study aimed to replicate the association signals from BMI GWAS in individuals of European descent. The study indicated that SEC16 homolog B (*SEC16B*), transmembrane protein 18 (*TMEM18*), glucosamine-6-phosphate deaminase 2 (*GNPDA2*), brain-derived neurotrophic factor (*BDNF*), fas apoptotic inhibitory molecule 2 (*FAIM2*), and melanocortin 4 receptor (*MC4R*) loci are not only associated with BMI in European ancestry individuals but also with obesity in Japanese ancestry individuals. On the other hand, 16 obesity-related SNPs could not be replicated in this study, supporting the heterogeneity of genetic susceptibility to obesity among various genetic ancestries. For instance, in contrast to the European cohorts, genes such as phosphotriesterase related (*PTER*) and secretogranin III (*SCG3*) were monomorphic for the respective variants in the studied Asian cohort. One of the genes whose polymorphisms were replicated in this study was *SEC16B*. *SEC16B* encodes long (Sec16L) and short (Sec16S) proteins required for mammalian cells to deliver intracellular substances from the endoplasmic reticulum to the Golgi apparatus (Watson et al., 2006; Bhattacharyya et al., 2007). Although Schmid et al. (2012) showed that *Sec16b* has the highest expression in subcutaneous adipose tissue and the lowest expression in the hypothalamus, Hotta et al. (2009) proposed that *Sec16b* expressed in the hypothalamus might be affecting energy regulation. *SEC16B* is not only an obesity susceptibility locus in individuals of Asian and European ancestry but also is related to BMI in individuals of African ancestry (Sahibdeen et al., 2018). Also, the polymorphism of *Tmem18*, which is highly expressed in the hypothalamus (Schmid et al., 2012), is one of the BMI-related loci being robustly replicated in individuals of Asian ancestry. A study focusing on *Tmem18* expression in the hypothalamic nucleus showed that *Tmem18*-deficient mice gain body weight compared with a control mouse, especially in males under a strict high-fat diet (Larder et al., 2017). Overexpression of *Tmem18* in hypothalamic paraganglia may affect food intake, increase energy expenditure, and reduce systemic fat and body weight.

Another gene highly expressed in the hypothalamus is *GNPDA2*. Genetic variants in or near *GNPDA2* have been shown to be associated with obesity in Asians (Hong et al., 2013), Pima Indians (Muller et al., 2019), and Europeans (Willer et al., 2009). In contrast, there are controversial data in childhood obesity; it has been shown that the *GNPDA2* locus is associated with BMI in a cohort from Mexico (León-Mimila et al., 2013), but not in an Asian cohort (Wang et al., 2012). *MC4R* is also a centrally acting gene known to be the most common cause of monogenic obesity in extreme childhood obesity. It is well recognized that hypothalamic pro-opiomelanocortin neurons regulate feeding behavior through the production of melanocortins and beta-endorphin from these neurons. MC4R is a major melanocortin receptor involved in regulating food intake and energy expenditure (Nogueiras et al., 2007). The *MC4R* has been reported as a risk gene associated with extreme obesity in adolescence and adulthood (Chambers et al., 2008; Hotta et al., 2009; Tenesa et al., 2009; Thorleifsson et al., 2009). Short-term administration of an MC4R agonist RM-493 increased individual resting energy expenditure and limited fat oxidation in obese individuals (Chen et al., 2015). However, two other clinical studies using MC4R agonists did not show any effects of regulating body weight (Krishna et al., 2009; Royalty et al., 2014). Further studies are needed in other to clarify the controversial findings reported.

In 2010, Ng et al. (2010) carried out a replication study of 12 BMI-associated loci from a European ancestry GWAS in a Chinese cohort. Five loci located at or near *GNPDA2*, *BCDIN3* domain containing RNA methyltransferase (*BCDIN3D*), SH2B adaptor protein 1 (*SH2B1*), *FTO*, and potassium channel tetramerization domain containing 15 (*KCTD15*) seem to be related to BMI in Chinese individuals. Two of them, *SH2B1* and *KCTD15* polymorphism (rs7498665 and rs29941), were replicated for the first time in an Asian cohort. *SH2B1* encodes SH2B adaptor protein 1, a member of the SH2-domain containing mediators family. It is expressed in both central and peripheral tissues (Ren et al., 2007). A study showed that central *Sh2b1* controls glucose homeostasis and insulin sensitivity (Duan et al., 2004) as well as hypothalamic leptin sensitivity (Ren et al., 2007). Peripheral *Sh2b1* regulates insulin sensitivity and glucose metabolism (Ren et al., 2007) whereas hepatic *Sh2b1* regulates lipid metabolism, particularly triacylglycerol and very-low-density lipoprotein content in the liver (Sheng et al., 2013).

The function of the *KCTD15* is still unknown. However, it has been shown that *Kctd15* deficiency resulted in a slow-growth/small size phenotype in zebrafish (Heffer et al., 2017). Particularly, the Kctd15 likely acts through interaction with adipocyte protein 2 (AP-2) (Liu et al., 2013), which is a critical regulator in adipogenesis (Shan et al., 2013), suggesting a possible molecular basis for the observed associations of *KCTD15* variants with obesity.

In 2012, Dorajoo et al. (2012) performed a BMI GWAS meta-analysis in Asian ancestry individuals (Singaporean, Malay, and Asian-Indian) and, among others, confirmed the relevance of the *FTO* locus. The authors replicated 13 loci which have been previously reported in European ancestry cohorts and found three novel variants (rs2287019, rs2241423, rs516175) associated with BMI in their Asian cohort. Interestingly, 16 loci previously found in the European ancestry GWAS were not associated with BMI in this study, possibly due to the genetic heterogeneity between present-day Asian and European ancestries. Rs2287019 variant maps in the vicinity of the *GIPR*, the gene encoding a G protein-coupled receptor for a gastric inhibitory polypeptide, which is strongly expressed in pancreatic beta cells (Saxena et al., 2010). It is involved in the incretin effect and in early pathophysiologic pathways that could lead to impaired glucose tolerance and T2D in humans. *Gipr*-deficient mice are more resistant to obesity after a high-fat diet (Miyawaki et al., 2002), which is likely due to the interplay of enhanced insulin sensitivity and inhibition of GIP signaling pathways in adipose tissue (Joo et al., 2017). *MAP2K5*, mitogen-activated protein kinase kinase 5, which is the closest gene to the BMI-related loci (rs2241423), plays a crucial role in the MAPK signaling pathway. Chen et al. (2014) showed that MAP2K5 is regulated by mir-143 and affects lipogenesis. Methionine sulfoxide reductase A, *MSRA*, located near the previously mentioned variant rs516175, regulates glucose metabolism and insulin response in mitochondria and has protective effects on insulin sensitivity in obese mice (Hunnicut et al., 2015). It is also a target of miR-193b which stimulates reactive oxygen species signal transduction and regulates lip sarcoma cell survival and adipose tissue–derived stromal/stem cells cell differentiation (Mazzu et al., 2017).

In 2012, a two-stage GWAS (Okada et al., 2012) in an East Asian cohort discovered two novel loci, nearby CDK5 regulatory subunit associated protein 1 like 1 (*CDKAL*) and kruppel like factor 9 (*KLF9*), which were associated with BMI. The study clearly implicated ancestry-specific effects of the *KLF9* locus, which was not found in previous analyses in European individuals (Speliotes et al., 2010), despite the sufficient statistical power to detect the locus based on the assumption of the same effect size and allele frequencies (MAF_Eur_ = 0.5, MAF_Asi_ = 0.4). A three-stage meta-analysis of eight BMI GWAS was performed, with the second phase being computer replication and the third phase being a *de novo* replication study (Wen et al., 2012). The analysis resulted in 10 loci reaching associations at genome-wide significance (*p* < 10^–8^). Seven of the ten loci are at the *FTO*, *SEC16B*, *MC4R*, *GIPR/*glutaminyl-peptide cyclotransferase like (*QPCTL*), adenylate cyclase 3 (*ADCY3*), *BNDF*, and *MAP2K5*, which have been previously shown to be associated with BMI in European ancestry individuals. Three novel loci in or near cyclin-dependent kinase 5 (*CDKAL1*), proprotein convertase subtilisin/kexin type 1 (*PCSK1*), and glycoprotein 2 (*GP2*) associated with BMI in an East Asian cohort. Kim et al. (2013) identified the prospero homeobox 1 (*PROX1*) locus in a GWAS for BMI in a cohort from Mongolia and replicated it in a cohort from Korea. However, the associations only reached suggestive significance with *p* < 10^–7^. The limited statistical power was likely attributed to the relatively small sample size (*n* = 1301). Albeit not statistically significant at the genome-wide level, the study also suggested protein tyrosine phosphatase receptor type D (*PPTRD*) and reelin (*RELN*) to be potential candidate genes that may have a role in the development of obesity.

In 2014, a two-stage GWAS (Wen et al., 2014) including 82,438 East Asian and 4301 South-East Asian individuals in the discovery and 47,352 East Asian individuals in the replication stage indicated four novel BMI-related loci reaching a significant level of genome-wide association: these loci in or near potassium voltage-gated channel subfamily Q member 1 (*KCNQ1*), aldehyde dehydrogenase 2 family member (*ALDH2*), inter-alpha-trypsin inhibitor heavy chain 4 (*ITIH4*), and 5′-nucleotidase cytosolic II *(NT5C2*).

*KCNQ1* variant (rs2237892) was initially reported in GWAS of T2D in Asian cohorts (Unoki et al., 2008; Yasuda et al., 2008), followed by replication reports in European cohorts (Unoki et al., 2008; Voight et al., 2010). Moreover, *KCNQ1* locus has been shown to be associated with waist circumference (WC adjusted for BMI) in an Asian cohort (Graff et al., 2017). KCNQ1 is expressed in islet cells and has been implicated in the regulation of insulin secretion (Ullrich et al., 2005).

*ALDH2* polymorphism rs671 is not only related to obesity but also to multiple complex traits such as drinking behavior (Jorgenson et al., 2017), triglycerides (Tan et al., 2012), and blood pressure (Feitosa et al., 2018). As suggested by Akiyama et al. (2017), the *ADH-ALDH* gene family may have a greater significant impact on BMI in East Asian individuals. Recent studies (Yu et al., 2016) suggested that ALDH2 is a positive regulator of adipocyte differentiation through the interaction with its upstream regulatory factor protein kinase C mediated by peroxisome proliferator-activated receptor gamma transcriptional activity. *ITIH4* is widely distributed in the blood and liver (Cai et al., 1998). The gene locus has been associated with schizophrenia (Goes et al., 2015) and blood serum protein levels in several GWAS (Emilsson et al., 2018). Early studies (Fujita et al., 2004) suggested that *ITIH4* locus is also associated with hypercholesterolemia in a Japanese cohort. *NT5C2* encodes a downstream cytosolic hydrolase that plays a considerable role in cellular purine metabolism by acting primarily on inosine 5′-monophosphate and other purine nucleotides (Novarino et al., 2014).

In 2017, Akiyama et al. (2017) implemented, so far, the largest imputation-based GWAS in 158,284 East Asians. They reported 112 BMI loci, 61 of which were novel, and pointed out that BMI-related loci are most likely shared among different ancestries; however, the effects of particular loci on BMI may vary among genetic ancestries.

## Comparison of BMI Susceptibility Loci Between European and Asian Ancestries

Most BMI-associated loci initially uncovered in studies with individuals of European ancestry have been widely replicated in Asian individuals (Frayling et al., 2007; Hotta et al., 2008; Cho et al., 2009; Yajnik et al., 2009; Dong et al., 2011; Moore et al., 2012; Vasan et al., 2012; Monda et al., 2013).

By reviewing all current BMI-related studies in Asian cohorts (Table 1), we found 92 loci (Supplementary Table S1) and compared them with GWAS in European cohorts. Forty-two of 92 BMI-related loci have been previously reported in European cohorts with *p* < 5 × 10^–8^ and had a consistent direction of effect on BMI. For the remaining 50 BMI-related loci, we observed no compelling evidence of replication (Supplementary Table S1). According to our defined criteria (*p* < 5 × 10^–8^ for GWAS, *p* < 0.05 for replication), the replications failed in the following cases: (1) 6 of 50 SNPs reached the genome significant *p*-value (*p* < 5 × 10^–8^) in Asian cohorts but not in European cohorts. Because it is unlikely that limited statistical power due to small sample size and minor allele frequencies would be a crucial factor (see Supplementary Table S1) explaining the failed replications, other reasons such as genetic heterogeneity or distinct phenotypic expression in different genetic ancestries may be considered. Exemplarily, East Asians showed a lower mean BMI (22.7 ± 3.59 kg/m^2^) (Okada et al., 2012) than European cohorts (27.24 ± 3.9 kg/m^2^) (Speliotes et al., 2010). (2) Twenty-three of 50 SNPs had a genome-wide significant *p*-value (*p* < 5 × 10^–8^) in Europeans, but no significant associations (*p* > 0.05) in Asian populations. Eighteen of these 23 variants were only directionally consistent but not significantly associated with BMI, and the remaining five SNPs were neither directionally consistent nor statistically significantly associated with BMI (*p* > 0.05) in Asian individuals. Limited statistical power could be a likely explanation for this observation. Compared with some large-scale studies in European cohorts with sample sizes ranging from 100,000 to 700,000, these Asian studies that failed to replicate the 23 variants were relatively small (1000 to 10,000). Two of the 23 loci non-replicated variants may have been due to marked differences in MAF between European and Asian individuals, as the MAF of rs17381664 and rs925946 were 0.002 and 0.06, respectively, in Asian individuals, and 0.37 and 0.29 in Europeans. Another possibility to be taken into account could be different causal variants between Asian and European individuals, resulting in a weak LD pattern in the Asian cohort, consequently leading to a weaker correlation between causal variants and marker SNPs. (3) For the remaining 21 SNPs, there was no convincing evidence for association with BMI in any of the two populations.

**FIGURE 1:**
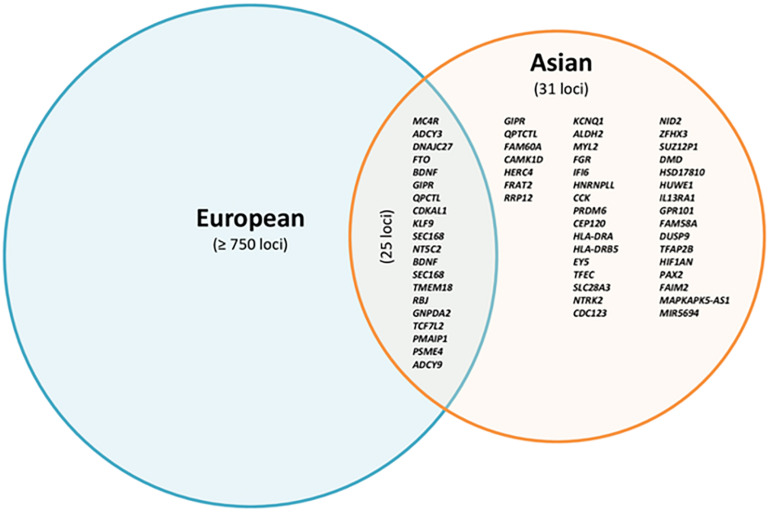
Overlap of reported loci associated with BMI in European and Asian cohorts based on *p*-value < 10^–8^.

In summary, the majority of BMI-associated loci overlap between studies in Asian and European cohorts with regard to the respective risk alleles, although their frequencies may slightly vary. We found 82 BMI susceptibility loci (results not shown) by screening associations with a *p*-value < 10^–8^ in individuals from Asia, after pruning by checking linkage disequilibrium (LD) through LD proxy module in a public LD online database from the National Institutes of Health (Division of Cancer Epidemiology & Genetics, 2020). We finally found 31 BMI loci that have only been associated with Asian cohorts (Figure 1 and Table 2). Of these 31 loci, two of them were monomorphic in European subjects (eyes shut homolog (*EYS*)—rs148546399 and nidogen 2 (*NID2*)—rs75766425) and eight were rare mutations [FGR proto-oncogene (*FGR*)—rs2076463, heterogeneous nuclear ribonucleoprotein L like (*HNRNPLL*)—rs77489951, cholecystokinin (*CCK*)—rs8192473, transcription factor EC (*TFEC*)—rs143665886, *LOC102724612*—rs77636220, FRAT regulator of WNT signaling pathway 2 (*FRAT2*)/ribosomal RNA processing 12 homolog (*RRP12*)—rs12569457, fibroblast growth factor receptor 2 (*FGFR2*)—rs1907240, and *ALDH2*—rs7305242]. *NID2*, *FGFR2*, and *ALDH2* had already been reported in the latest T2D GWAS in an East Asian cohort (Spracklen et al., 2020). On the other hand, five loci were monomorphic or rare in Asian cohorts [*FTO*—rs9930333, LDL receptor related protein 1B (*LRP1B*)—rs2890652, cell adhesion molecule 2 (*CADM2*)—rs13078807, solute carrier family 39 member 8 (*SLC39A8*)—rs1310735, and protein kinase D1 (*PRKD1*)—rs11847697]. These variants of the 31 loci associated with GWAS in Asian cohorts explained only 0.926% of the phenotypic variance (Table 2).

We also integrated four GWAS works (Kang et al., 2010; Ng et al., 2012; Salinas et al., 2016; Chen et al., 2017) (Supplementary Table S2) conducted in African ancestry individuals (including African American and Afro-Caribbean and sub-Saharan African). Only one novel SNP (rs80068415) reached a GWAS significant threshold and five novel loci showed suggestive association with BMI at *p* < 1 × 10^–5^ in the studied African cohorts (Supplementary Table S3). The variant (rs80068415) identified in this GWAS only explained 0.065% of the variance (Supplementary Table S3). Because of LD patterns, six loci contained two SNPs in high LD with previously identified index SNP related to BMI with *p* < 5 × 10^–8^ in a European cohort. The variant (rs2033195) was in high LD with rs10055843 (*r*^2^ = 0.96) which are associated with BMI at *p* = 7.2 × 10^–18^ in European individuals. The variants rs815611 and rs1346482 were in high LD (*r*^2^ = 0.92), and the latter was associated with BMI (*p* = 2 × 10^–19^) in European cohorts. The underlying susceptibility locus of potentially African-specific rs80068415 is located in the region of semaphoring-4D (*SEMA-4D*). The proposed mechanism behind *SEMA-4D* on obesity is likely complex and may be mediated through regulatory multiple biological processes. Obesity usually follows a chronic inflammatory condition and T-cell accumulation has a positive correlation with adiposity. SEMA-4D seems to be a key player in the activation and differentiation of T cells and SEMA-4A could promote T helper 1 (Th1) cell differentiation (Worzfeld and Offermanns, 2014). This novel variant (rs80068415) seems to be highly specific to Africans as it is monomorphic in other populations.

The *FTO* locus manifests the strongest association signal with obesity in both Asian and European populations. Although the effect direction is consistent, the number of genetic variants varies between the populations. Nineteen *FTO* variants reached a genome-wide significance level for association with BMI in Europeans, whereas only four variants were associated with Asians. For instance, the top BMI-associated *FTO* signal found in GWAS in European individuals was rs1558902 (*p* = 4.8 × 10^–120^), whereas rs11642015 (*p* = 2.04 × 10^–81^) was the prominent hit in the Asian population. It is evident that differences in genetic architecture (e.g., rs9930333 with *p* = 10^–103^ is the only polymorphism in Europeans) and evolutionary selective pressure (Liu et al., 2015) may at least partially explain the observed differences in associations at the variants level. However, it is worth mentioning that GWAS in Asian cohorts have emerged recently and genetic association studies have mostly focused on replication of previously reported signals from other GWAS. Furthermore, the reported studies in Asian cohorts are limited by a relatively small sample size compared with studies in European cohorts. Nevertheless, there is an enormous potential for large-scale genome-wide studies in cohorts of Asian ancestry, which may lead to the identification of novel players in the genetic architecture of human obesity.

**FIGURE 2:**
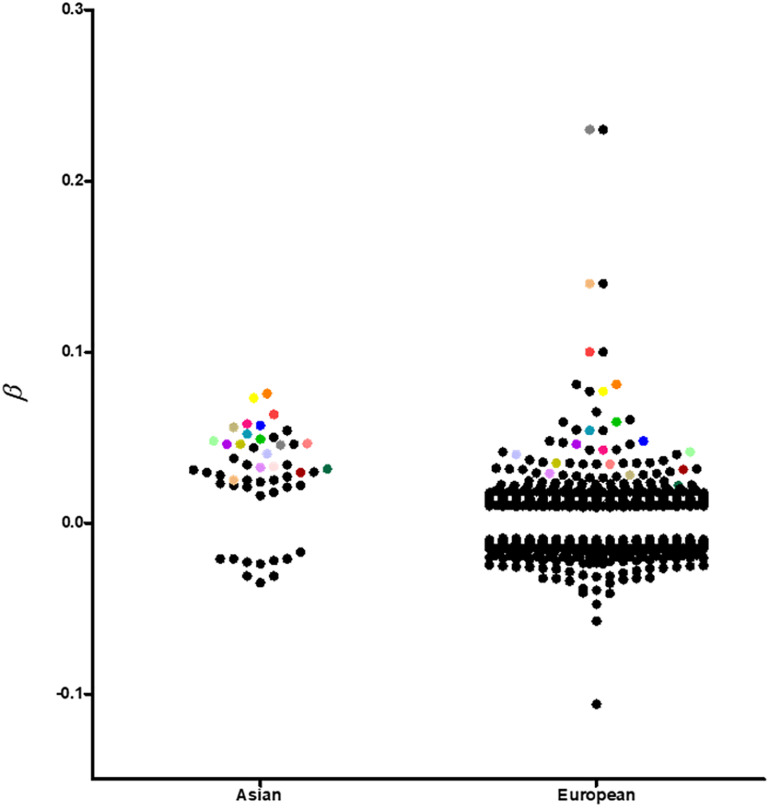
A visual representation of all replicated and non-replicated loci at *p*-value < 10^–8^ in GWAS conducted in individuals of European and Asian ancestries. Replicated loci are coded with the same color.

**FIGURE 3:**
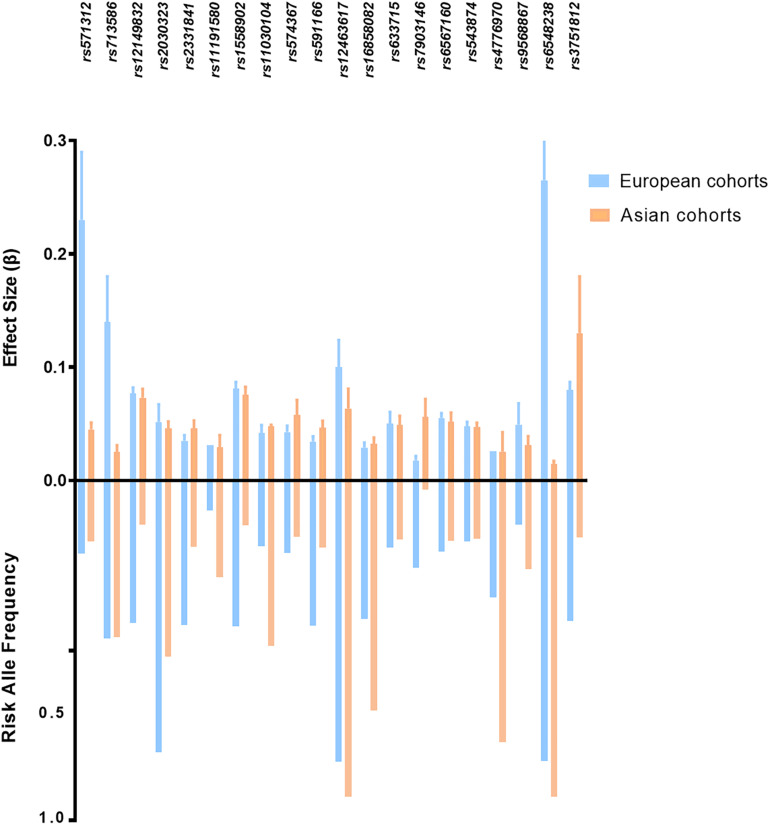
Risk allele frequency and effect size of top ranked obesity susceptibility loci which reached genome association significant *p*-value in European and Asian cohorts. BMI-related loci shown in this figure with *p* < 5 × 10^–8^ in both cohorts. *p*-values and effect size are according to reference studies reported (Supplementary Table S1). The allele frequency is based on genome Aggregation Database (gnomAD).

Although most of the BMI associated loci showed consistent effect directions between Asians and Europeans (Figure 2), the sample effect sizes differ substantially. The frequency of risk alleles varies from 1 to 40% between the genetic ancestries. For instance, the allele frequency of the *FTO*-rs12149832 obesity risk alleles differs by 40%, whereas the effect size on BMI is comparable. In contrast, the frequency of variants in *MC4R* (rs571312) or *ADCY3/DNAJC27* (rs713586) in the European populations is similar to that in the East Asian populations based on genome Aggregation Database (gnomAD), but the difference in effect size on BMI accounted for about 20% (Figure 3 and Supplementary Table S1).

## Copy Number Variations in Obesity in Asian Cohorts

Along with the SNPs, copy number variations (CNVs), which are not only abundant in the human genome (Tuzun et al., 2005) but also have a vital influence on gene expression (Stranger et al., 2007), have emerged as another critical genetic label continuously attracting researchers’ attention in the field of complex polygenic traits. Their “gene dosage” effect mediates the risk or protection against human diseases such as obesity (Jacquemont et al., 2011). One of the important CNVs related to BMI in the Asian population was reported on 10q11.22 (Sha et al., 2009). Pancreatic polypeptide Y receptor Y4 (*PPYR1*) located under this CNV area appears to be a plausible gene eventually related to obesity, and Shebanits et al. (2018) have also found similar findings in a Swedish cohort which suggested an association of *PPYR1 (NPY4R)* with WC in women. PPYR1 is one of the receptors of pancreatic polypeptide (PP), and several studies demonstrated that PP regulates the food intake via PPYR1 (Batterham et al., 2003). Yang et al. (2013) confirmed the association of a CNV on 16p12.3 with obesity-related phenotypes in a European but not in Asian cohort and suggested G protein-coupled receptor class C group 5 member B (*GPRC5B*) as a candidate obesity gene mapping within this chromosomal region. The authors emphasized the necessity of considering various ancestries in genetic association studies, particularly CNVs, which are characteristic for their considerable variation across genetic ancestries. Sun et al. (2013) tested eight CNVs (2p11.2, 10q11.22, 11q13.4, 16p11.2, 5p15.33, 15q11.2, 8q24.3) in young Chinese subjects, but proved only CNV 8q24.3 being associated with obesity, whereas no significant association was found for the other seven CNV candidates. *BAI1*, brain-specific angiogenesis inhibitor 1, which is located within the CNV 8q24.3, is postulated to be a member of the secretin receptor family and is the only member in its family transcriptionally regulated by p53 (Van Meir et al., 1994). Zhang et al. (2015) replicated three obesity-related loci 10q11.22, 4q25, and 11q11 in Han Chinese children and noted the strong cumulative effect of these loci on the risk of obesity. Furthermore, they also pointed out a significant interplay between CNVs (10q11.22) and dietary behaviors (meat-based). On the other hand, the salivary amylase gene (*AMY1*), whose copy number has been positively correlated with salivary amylase protein level (Perry et al., 2007), has delivered rather inconsistent findings concerning obesity. Perry et al. (2007) suggested that more *AMY1* copies exist in populations with high-starch diets than in those with traditionally low-starch diets. This points to a restricted selection of *AMY1* copies through a dietary shift early during human evolutionary history, especially in some ethnic groups such as East Asians known to prefer high-starch diets. However, a recent study failed to support the association of *AMY1* and *AMY2A* CNVs with obesity in two East Asian cohorts (Yong et al., 2016). A similar conclusion was drawn by Usher et al. (2015) who did not find any association of *AMY1* CNVs with obesity or BMI in a study including three European cohorts. Nevertheless, despite lacking evidence of an association between *AMY* CNVs and obesity, these studies inspired and promoted a novel perspective for future genetic association studies for obesity whereby variation in diet or environment exposures and their interaction with our genomes need to be considered.

## Future Perspectives

### Gene × Environment Interaction

Obesity is a complex disease affected by both environment and genes. Because of increasing globalization, urbanization, and improved economic status, human diet structure and life habits have changed in Asia. Precisely, it has been observed that increased availability of food, better transport facilities, better healthcare facilities, reduced physical activity by mechanization, preference of viewing TV and videos (sedentary style), and increased use of automobiles and these changes in their life habits are associated with increased obesity prevalence in urban and rural populations, particularly in developing countries. Moreover, it is important to emphasize that there are also changes in their diet structure, such as a tendency to eat more finely processed carbohydrates (such as rice) and fat-rich items (Ramachandran et al., 2012). While the societal scale environment could cause the obesity epidemic, it is also known that genetic differences underlie the variation in BMI between individuals and that gene × environment interactions may be important in this context. A recent study concluded that nutrition has the strongest environmental effect on obesity risk at the *FTO* locus. Using genetic, anthropometric, and lifestyle variables collected as part of the UK Biobank, they assessed gene-by-environment interactions and how they modify the effect of *FTO* variants on BMI. The authors reported significant interactions between rs1421085 and a number of lifestyles and environmental factors, including alcohol, consumption, and mean sleep duration, with overall diet having the strongest effect on modifying *FTO* risk (Young et al., 2016). There is no doubt that gene–environment interactions are necessary to be understood to explain the underlying pathophysiology of obesity as a complex disease across the genetic diversity present in contemporary individuals across the globe.

### Rare Genetic Variants

The loci associated with obesity remain to be further investigated, as the currently known loci only explain a small fraction of the variation in obesity and its measures such as BMI. Whereas common polymorphisms have been the main target of the majority of large-scale genetic studies so far, rare genetic (low frequency) variants with significant effects may substantially contribute to our understanding of the genetic heterogeneity of obesity and fat distribution. In this regard, further intensive research is inevitable in the cohorts of Asian ancestry to identify novel obesity loci either specific in Asian ancestry or common for various ancestries and thus provide new insights into the mechanisms underlying obesity.

### Understanding the Functional Consequences of Obesity-Associated Variants

The function of most of the genes within obesity-associated loci remains to be clarified. Although numerous polymorphisms associated with obesity have been revealed so far in studies including various ethnicities, identification of the respective target genes of these variants remains challenging. This is mostly attributed to the variety of regulatory mechanisms SNP may be involved in, which makes it difficult to predict the most likely target gene. While in most cases, these genes map in close vicinity of their functional variant, they may also be positioned hundreds of kilobases upstream or downstream of the genes.

In line with this, it has to be noted that most of the genes reported in this review are based on the “closest” gene approach, which admittedly is not a highly accurate approach. Although it may be true for some obesity loci (e.g., FTO), for most of the currently known obesity susceptibility loci, no target genes of the associated genetic variants have been robustly validated. Instead, the closest or nearby genes are being reported and proposed as potential candidate genes explaining the observed associations.

### Measures of Obesity

The classical and mostly applied measure of obesity is BMI. However, because of differences in phenotypes and body composition in Asian and European populations, BMI may not be the most appropriate measure to assess the degree of obesity globally. This phenomenon may cause GWAS to miss important genetic variants in specific populations or subgroups. At the same time, inaccuracy in the measured phenotypes may result in false-positive association signals. Establishing new tools/measures including whole-body MRI scan and body composition techniques to easily and quickly assess obesity will be inevitable to refine and make the search for obesity-related genes more efficient.

### Fine Mapping in Multi-Ethnic/Trans-Ethnic Studies

A growing number of multi-ethnic/trans-ethnic studies have been completed in populations of non-European ancestry in addition to replication studies in recent years. The potential ability to use trans-ethnic studies is identifying common genetic variants shared across different ancestries, as well as ancestry-specific disease predisposing variants, and interactions between genetic variants and the environment that can be shared or ancestry specific as well. Moreover, the diversity of LD patterns across various genetic ancestries can be leveraged to indicate causal variants. Moving beyond GWAS, also other approaches such as fine mapping studies are a valuable attempt to apply to multi-ethnic cohorts to get a better understanding of the role of novel loci implicated in obesity.

Fine-mapping strategies typically follow the GWAS findings aiming at prioritization of variants within susceptibility regions in the genome. Although the original GWAS can suggest a region that is likely to include a causal variant, additional strategies (fine mapping, whole-exome, and whole-genome sequencing) are necessary to distinguish most likely functional variants from only correlated causal variants. A major challenge in identifying underlying causal SNPs are the presence of LD, which can lead to highly correlated association results and multiple significant SNPs at a locus of interest. Most of the GWAS performance so far assume association analyses in relatively homogenous populations with consistent patterns of LD; this is straightforward for discovering associated variants. However, it can be challenging in multi-ethnic studies, where distinguishing multiple nearly equivalent variants may need hundreds of thousands of individual samples. Fine mapping in different ancestries is a method of lessening the barrier of LD and aids this process by selecting and prioritizing variants most likely responsible for complex traits. In addition, trans-ethnic fine mapping is a powerful approach for both narrowing the underlying causal variants in known loci as well as in discovering novel variants for complex traits (Zaitlen et al., 2010). Fine mapping in populations with relatively limited LD patterns like in individuals of African (Guo et al., 2013) or Asian (Hotta et al., 2008) ancestry may be helpful in the dissection of genetic architecture within a population and in pinpointing the causal variant. In the future, more trans-ethnic fine mapping studies will be inevitable in dissecting the genetic architecture of complex traits such as obesity. Considering that many complex traits are driven by large numbers of variants of small effects, which likely interact with the environment in complex ways, detailed mapping of genetic architecture regulatory networks and G × E effects will be an essential task for fully understanding human disease biology (Boyle et al., 2017).

## Conclusion

In summary, GWAS has exhibited a large number of BMI-associated loci over the past decade, providing an effective way to understand better obesity mechanisms which are essential on our way to improve the treatment of obesity. Although the pioneering large-scale GWAS were mostly conducted on individuals of European ancestry, there has been remarkable progress, which is now closing the gap between our knowledge of obesity genetics in European versus Asian ancestries. It should be noted that GWAS executed in Asian cohorts have not only affirmed the potential role of previously associated obesity loci but also displayed novel ones, which have been missed in the initial genetic studies in individuals of European ancestries. In addition, follow-up GWAS research strategies in multi-ethnic/trans-ethnic studies are worthwhile to conduct. At last, despite a large number of currently known obesity risk loci, the molecular mechanisms underlying this complex disease are not fully explained yet, and neither is the variation across human diversity in terms of obesity.

## Author Contributions

CS wrote the original draft of the manuscript. CS, EG-J, and PK reviewed and edited the manuscript. EG-J and PK supervised the study. All authors contributed to the article and approved the submitted version.

## Conflict of Interest

The authors declare that the research was conducted in the absence of any commercial or financial relationships that could be construed as a potential conflict of interest.
